# MRI abnormal patterns of lumbar paraspinal muscles in patients with amyotrophic lateral sclerosis and lumbosacral radiculopathy: a comparative study

**DOI:** 10.3389/fneur.2026.1751139

**Published:** 2026-03-17

**Authors:** Yuting Ren, Xu Han, Kang Zhang, Songtao Niu, Bin Chen, Xingao Wang, Fan Jian, Hua Pan, Zaiqiang Zhang, Xin Chen

**Affiliations:** 1 Department of Neurology, Beijing Tiantan Hospital, Capital Medical University, Beijing, China; 2 National Clinical Research Center for Neurological Diseases, Capital Medical University, Beijing Tiantan Hospital, Beijing, China; 3 Department of Radiology, Aerospace Center Hospital, Beijing, China; 4 Department of Radiology, Guangzhou First People's Hospital, School of Medicine, South China University of Technology, Guangzhou, Guangdong, China

**Keywords:** amyotrophic lateral sclerosis, fatty infiltration, lumbosacral radiculopathy, magnetic resonance imaging, paraspinal muscles, relative cross-sectional area

## Abstract

**Background:**

Recent evidence highlights the potential predictive value of paraspinal muscle degeneration in amyotrophic lateral sclerosis (ALS). However, the magnetic resonance imaging (MRI) characteristics of degeneration in lumbar paraspinal muscles in ALS and lumbosacral radiculopathy (LR) remain unclear.

**Methods:**

Comparison of fatty infiltration (FI) and relative cross-sectional area (rCSA) of the paraspinal muscles was conducted between 38 ALS patients and 32 LR patients.

**Results:**

The mean rCSA of the multifidus (MF), erector spinae (ES), and psoas major (PM) muscles was lower on the symptomatic onset side compared to the contralateral side at the L3–L5 segments in patients with ALS. On the symptomatic onset side, the FI of the ES (L1-L4 segments), MF (L4 segment), and PM muscles (L1, L2, and L4 segments) was significantly higher in ALS patients who had pathological spontaneous activity (PSA) than in those without PSA. At the L3-L5 segments on the symptomatic onset side, the mean rCSA of the MF, ES, and PM muscles was significantly higher in LR patients compared to ALS patients (*p* < 0.01). Similar differences in the rCSA of the MF, ES, and PM muscles were observed between lower limb-onset ALS patients and LR patients (*p* < 0.05). In addition, mild associations were observed between declines in the ALS functional rating scale (ALSFRS)-lower score and decreases in the rCSA of MF and PM muscles, as well as increased FI of the MF and ES muscles.

**Conclusion:**

The decrease in the rCSA of the paraspinal muscles on the symptomatic onset side suggests progressive involvement of muscle fibers in ALS patients. The presence of PSA in the paraspinal muscles appears to be more valuable and sensitive for evaluating fatty substitution than muscle atrophy in ALS. MRI parameters of the paraspinal muscles may be useful for monitoring disease progression in ALS and distinguishing ALS, especially lower limb-onset cases, from pauci-symptomatic LR.

## Introduction

Amyotrophic lateral sclerosis (ALS) is a progressive neurodegenerative disease characterized by degeneration of upper and lower motor neurons, leading to weakness of the bulbar, thoracic, and limb muscles. Muscle denervation is a major clinical feature of ALS and enables the investigation of patterns of disease spread *in vivo* (1). Several studies have examined the involvement of motor neuron groups innervating the paraspinal muscles in ALS (2–5); however, only a few studies have investigated paraspinal muscle magnetic resonance imaging (MRI) in ALS, and these have produced inconsistent conclusions (1, 6, 7). Jenkins et al. (1) reported that patients with motor neuron disease (MND) had higher relative T2 muscle signal than controls at baseline and higher T2 signal was associated with greater overall disability and clinical weakness. The Diamanti group confirmed that atrophy was more frequent on T1-weighted muscle MRI in ALS patients than in HCs and observed a trend toward agreement between MRI and clinic-EMG data for the paraspinal muscles (6). The same research team later verified that paraspinal T1-weighted MRI could help distinguish spinal ALS patients from both healthy and pathological controls (7). However, whether the degenerative changes are related to disease severity remains unclear. In this context, this study aimed to assess MRI-derived heterogeneity in ALS patients and pauci-symptomatic lumbosacral radiculopathy (LR) patients by comparing the patterns and severity of the involved paraspinal muscles.

## Methods

### Participants

In this cross-sectional pilot study, we enrolled 38 newly diagnosed probable or definite ALS patients, according to the El Escorial criteria (8), between 1 August 2020 and 31 June 2024, at the Department of Neurology, Beijing Tiantan Hospital. The onset site was classified as spinal in 35 patients (92.1%) and bulbar in three patients (7.9%). Disability was assessed using the ALS functional rating scale–revised (ALSFRS-R; 0–48), a validated 12-item instrument for monitoring disease progression in ALS patients (9). Each item was rated from 0 to 4 (4 indicating fully intact function and 0 indicating complete loss), with higher scores indicating better functional retention. The ALSFRS-R scores for the lower limbs, corresponding to items 7–9 (ALSFRS-lower, 0–12), were also recorded. The exclusion criteria were inability to provide informed consent, contraindications to MRI, ankylosing spondylitis, scoliosis, symptomatic lumbar spondylosis, lumbar spinal tumor, previous lumbar spine trauma or surgery, pregnancy, or respiratory failure impairing the ability to lie supine in the MRI scanner.

A total of 32 pauci-symptomatic LR patients were also recruited for MRI analysis. Pauci-symptomatic LR patients are defined as those with pathological evidence of compression or inflammation of the lumbosacral nerve roots, presenting only with motor symptoms such as muscle weakness, muscle atrophy, and diminished or absent tendon reflexes (10–13), while lacking classic features such as severe radiating pain, sensory changes, bowel or bladder dysfunction, gait disorders, and other functional impairment (14). Compression or inflammation of the lumbosacral nerve roots was identified based on MRI findings, lumbar puncture results, and EMG findings. Simultaneously, no progression of motor symptoms was observed in all LR patients, and no evidence of bulbar or upper limb involvement was found throughout the 2-year follow-up period. Demographic and clinical features of LR patients are summarized in Supplementary Table 1. Among the ALS and LR groups, the symptomatic onset side was defined as the side initially affected by motor deficits, as confirmed by EMG abnormalities. This study was approved by the medical ethics committee of Beijing Tiantan Hospital, and all participants provided written informed consent in accordance with the Declaration of Helsinki.

### Standard electrodiagnostic examination

Electromyography was performed using concentric needle electrodes, and a Medtronic Keypoint Net EMG system was employed in this investigation. Filter, gain, and sweep speed were set to 5 Hz–10KHz, 100 μV/div, and 20 ms/div, respectively. Skin temperature was monitored and maintained above 32 °C. Patients were examined at rest in a comfortable prone position. The posterior superior iliac spine line was used as a reference to identify the spinous process of the L5 segment. The levels of the T10-T12 and L4-S1 segments were then determined by palpation, moving cephalad and caudad from L5, respectively. Superficial and deep muscle regions were examined at the T10-T12 and L4-S1 thoracic and lumbar segment levels. Needles were inserted 1–2 cm lateral to the spinous process of these segments, at a right angle to the skin surface.

In each paraspinal muscle, spontaneous activity was assessed at four different sites, with three needle insertions performed in each of the standard four quadrants. Fibrillations (fibs) and positive sharp waves (PSWs) were considered present if reproducible trains lasting at least 1 s were observed following needle insertion in at least two different sites within a muscle or in a single site when confirmed on two additional repeat tests (15). A numeric grading system was used to semi-quantitate each of these spontaneous activities: +1, rare spontaneous potentials, recordable at one or two sites only after some searching, including insertional positive discharges induced by moving the needle electrode; +2, occasional spontaneous potentials, easily recorded at two or more sites; +3, frequent spontaneous potentials, recordable regardless of the position of the needle electrode; and +4, abundant spontaneous potentials, nearly filling the screen of the oscilloscope (16). Paraspinal muscle motor unit potential morphology and recruitment patterns were not considered in this study. The associations between active denervation in paraspinal muscle and clinical indicators were evaluated. All ALS and LR patients underwent nerve conduction and needle electromyography studies performed by the same neurophysiologist.

### Magnetic resonance imaging

Patients underwent a 3 T MRI (GE Medical Systems, USA) lumbar spine examination that included an axial TSE T2 sequence (slice thickness = 3 mm; TR/TE, 2132/120.20 ms; FoV = 200 mm; matrix = 512 × 512; bandwidth 25) centered on the spine, extending from the lumbar to the sacral region.

MRI data from all participants were collected and analyzed using a picture archiving and communication system (PACS). Muscle parameters were measured on the middle layer of the MRI at each segment (17). The psoas major (PM) muscle was also included in the evaluation; although it is not classified as a paraspinal muscle, it has similar features and functions.

### Image analysis and MRI data acquisition

**Figure 1:**
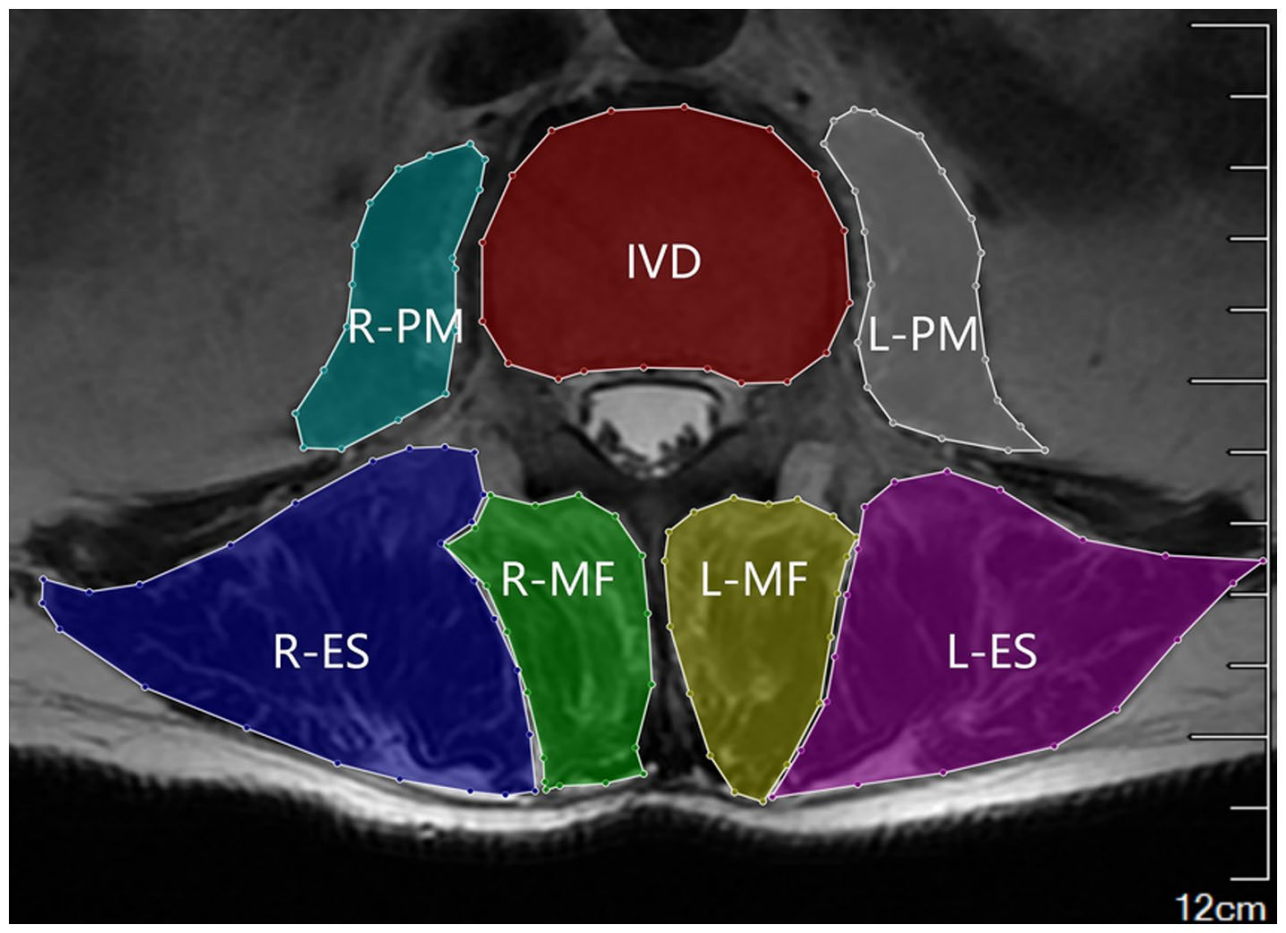
Contours of the muscles of interest, labeled using the LabelMe software.

The threshold method was used to measure the degree of fatty infiltration (FI) in paraspinal muscles. To improve the efficiency of data processing, a Python script was developed to calculate FI automatically. First, MRI images from the two groups (ALS and LR) were uniformly named using the format XX_N_L.png, where XX is the group code (ALS or LR), N is the index number of the images from the same patient within a group, and L is the lumbar segment number. In this study, five images were taken from the L1-L5 segments, so N∈ [1,5]. Then, the contours of the multifidus muscle (MF), erector spinae (ES), PM, and intervertebral disc (IVD) were labeled using LabelMe (Figure 1), an annotation software used for constructing artificial intelligence (AI) segmentation model datasets. The contour of each muscle of interest was stored as a series of points in a JSON file, which could be loaded by the Python script. The Python Imaging Library (PIL) module was imported in the Python script to draw masks of the muscles of interest based on the contour points from the JSON file.

MRI images were loaded as matrices of pixel grayscale values. A threshold was carefully selected to differentiate pixels representing normal muscle from those representing fatty tissue. In this study, a threshold value of 50 was selected to identify fatty tissue, meaning that any pixel with a grayscale value greater than 50 was considered to represent fatty tissue. This threshold was determined by manually checking and comparing subcutaneous fat on the images by imaging experts (X.C. and X.H.) who were blinded to the clinical status of each participant.

**Figure 2:**
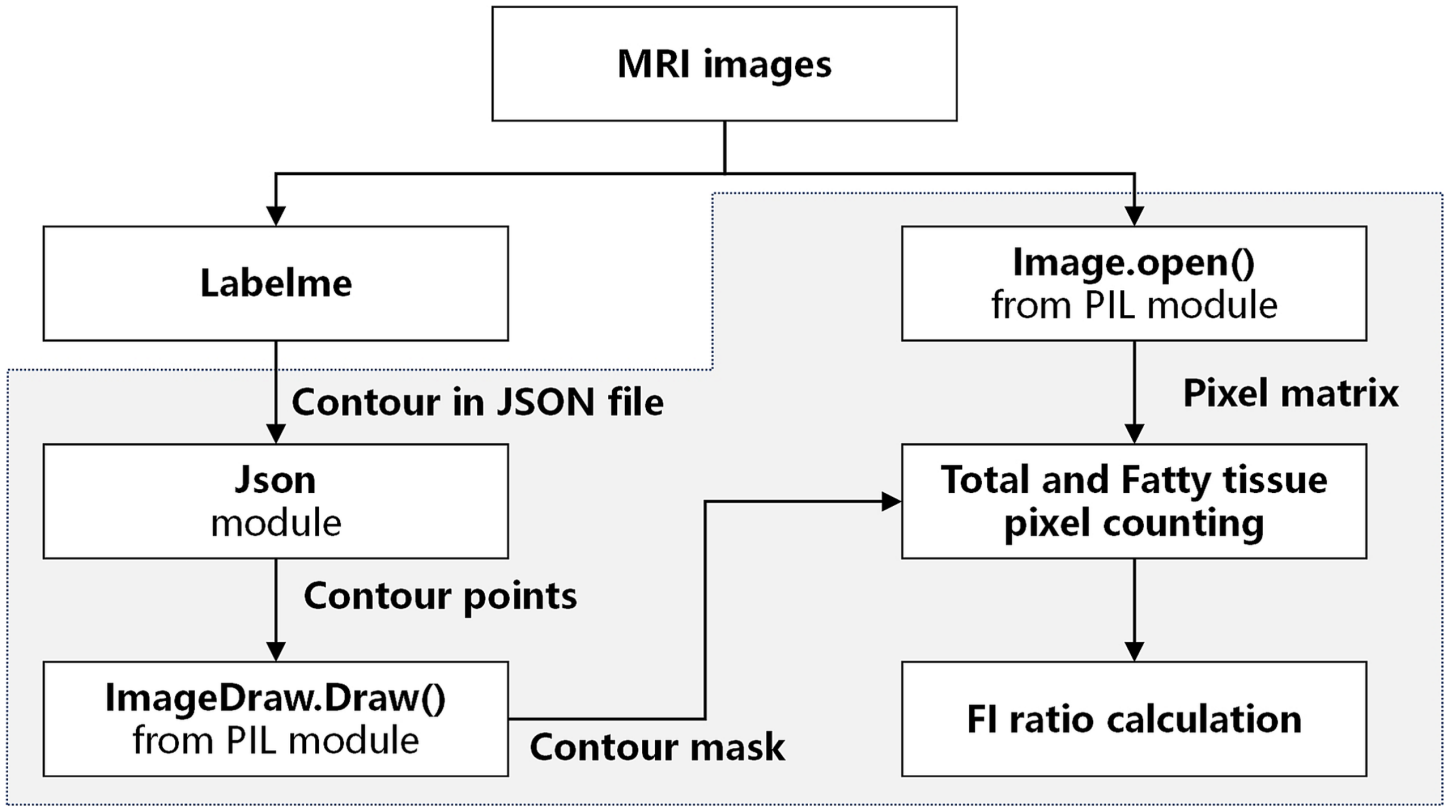
Image processing workflow. LabelMe is an open-source software commonly used as an annotation tool for AI segmentation model dataset construction. Steps highlighted in the grey area were performed using Python scripts.

Paraspinal muscle cross-sectional areas (CSAs) were measured using the grayscale discrimination method described by Ranson et al (18). CSAs were calculated by defining the region of interest (ROI) according to the boundaries of each paraspinal muscle in the cross-sectional images. The relative cross-sectional area (rCSA) is the ratio of the paraspinal muscle CSA to the CSA of the vertebrae of the same segment. This measure was adopted to eliminate individual differences in muscle volume that could affect the results (19). Using the muscle mask as a reference, the total number of pixels belonging to a specific muscle of interest was considered its CSA. Meanwhile, the number of pixels representing fatty tissue was counted and divided by the total number of pixels in the muscle to calculate the FI ratio. Image processing steps were performed as outlined in Figure 2. All parameters were validated independently by two neurologists specializing in neuromuscular disease (Z.Q.Z. and H.P.). The mean value of the two measurements was used for analysis.

### Statistical analysis

Statistical analyses were performed using SPSS Statistics (version 27; IBM Corp., Armonk, NY, USA). The normality of data was assessed using the Kolmogorov–Smirnov test. The independent-samples *t*-test and the Mann–Whitney U test were used to assess differences in numerical data, and a chi-squared test was used to compare categorical data between the two groups. Partial correlation analysis was used to explore the correlations between FI and rCSA with clinical variables, controlling for age and body mass index (BMI). Statistical significance was set at a *p*-value of < 0.05.

To evaluate the consistency and reproducibility of our data, the intraclass correlation coefficient (ICC) was used to assess inter-rater agreement. Intra-rater and inter-rater reproducibility of < 5% were first achieved before measurements were performed for the entire muscle.

## Results

### Participants

The ALS group included 16 female and 22 male participants, with a mean age of 55.4 ± 11.7 (30–77) years and an average BMI of 24.3 ± 2.9 (19.3–30.6). The LR group included 14 female and 18 male participants, with a median age of 61 years (interquartile range (IQR) 12) and an average BMI of 23.2 ± 4.1 (15.6–31.9). Age (*p* = 0.122), sex (*p* = 0.890), and BMI (*p* = 0.202) did not differ significantly between the ALS and LR groups. Clinical and demographic data are summarized in Table 1.

**Table 1:** Demographic and clinical features of patients with ALS and control subjects.

Item	ALS (*n* = 38)	LR (*n* = 32)	*p* value
Gender (M/F)	22/16	18/14	0.890
Age	55.4 ± 11.7	61.0 (12.0)	0.122
Disease duration	10.0 (11.3)	9.0 (20.3)	0.243
Age of onset	54.1 ± 11.8	57.1 ± 13.5	0.331
BMI	24.3 ± 2.9	23.2 ± 4.1	0.202
ALSFRS-R (0–48)	42.2 ± 2.6	–	–
ALSFRS-R-lower (0–12)	8 (4)	–	–
PSA (+/−)	27/11	20/12	0.071

ALS, amyotrophic lateral sclerosis; LR, lumbosacral radiculopathy.

Means ± standard deviation for normal distribution data.

Medians and interquartile ranges in brackets for abnormal distribution data.

*p* values indicate differences between patients and healthy control subjects calculated by using the *Mann–Whitney U* or *Fisher exact test*, as appropriate.

### Intra-group differences in FI and rCSA

**Figure 3:**
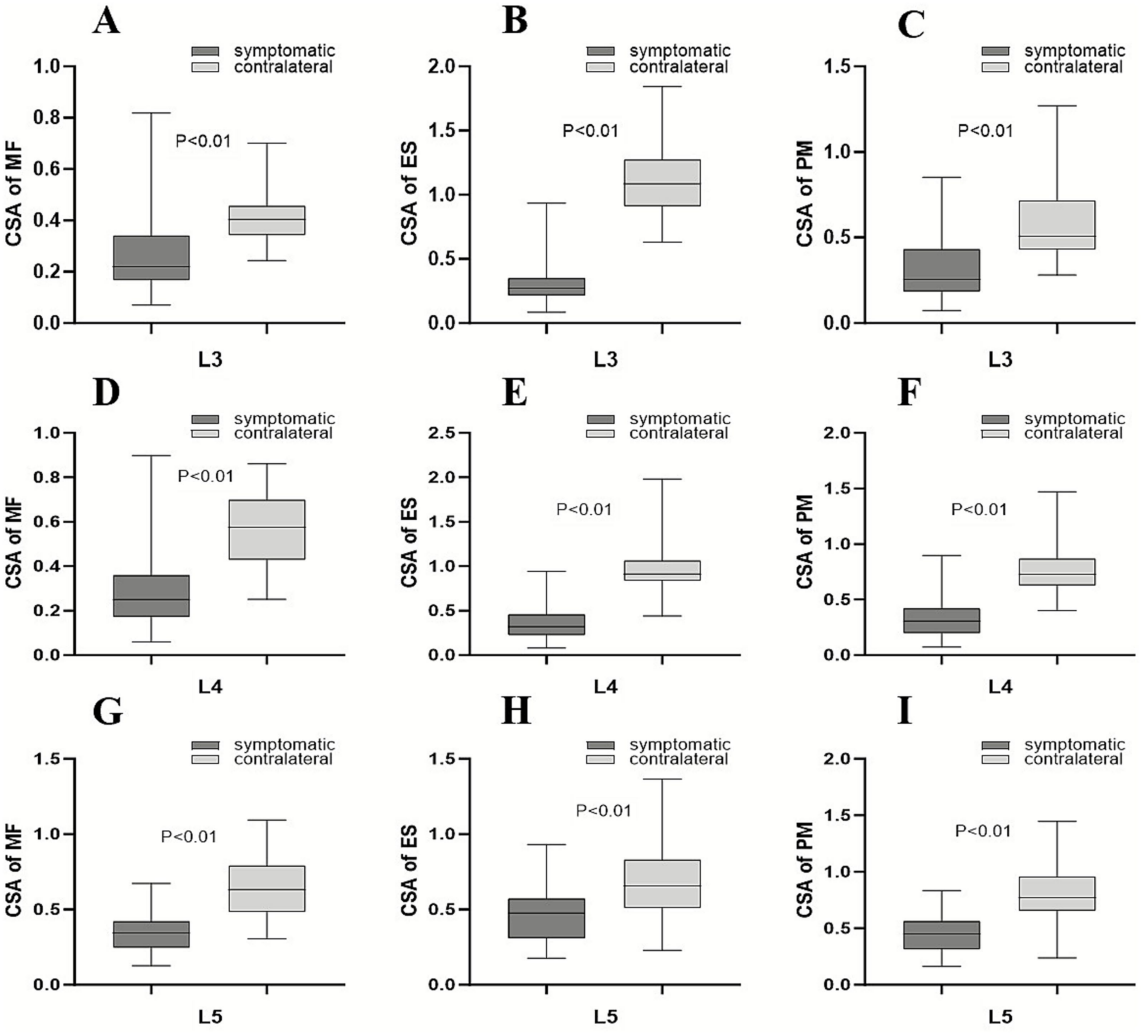
Box-and-whisker plots showing comparative analysis of the rCSA of the MF **(A,D,G)**, ES **(B,E,H)**, and PM **(C,F,I)** on the symptomatic onset and contralateral sides at the L3 **(A–C)**, L4 **(D–F)**, and L5 **(G–I)** segments in patients with ALS. The horizontal line within the box represents the median. The bottom and top edges of the box represent the 25th and 75th percentiles, respectively. The whiskers (error bars) represent the full range. Statistical significance was set at a *p*-value of < 0.05 for all analyses.

In ALS patients, the mean rCSA of the MF, ES, and PM was smaller on the symptomatic onset side than on the contralateral side at the L3-L5 segments (L3 segment: 0.22 (0.17) vs. 0.41 ± 0.10; 0.27 (0.14) vs. 1.09 ± 0.26; 0.26 (0.25) vs. 0.51 (0.29); L4 segment: 0.28 ± 0.16 vs. 0.58 ± 0.16; 0.37 ± 0.20 vs. 0.94 (0.28); 0.35 ± 0.19 vs. 0.77 ± 0.21; L5 segment: 0.34 ± 0.13 vs. 0.64 ± 0.19; 0.48 ± 0.19 vs. 0.69 ± 0.28; 0.46 ± 0.17 vs. 0.80 ± 0.27; all *p* < 0.01) (Figure 3). No differences were observed at the L1-L2 segments. In contrast, no significant differences were found in the LR group at the L1-L5 segments (Table 2).

**Table 2:** MRI data (FI and rCSA) of L1-L5 segments for ALS and LR patients.

Item	ALS (*n* = 38)		LR (*n* = 32)		*P*^1^value	*P*^2^value
	S	C	S	C		
L1						
MF FI	0.37 ± 0.19	0.40 ± 0.18	0.39 ± 0.16	0.41 ± 0.16	0.492	0.502
MF rCSA	0.29(0.12)	0.29(0.10)	0.26 ± 0.11	0.25 ± 0.11	0.494	0.466
ES FI	0.16 (0.16)	0.17 (0.15)	0.14 (0.14)	0.15 (0.17)	0.199	0.354
ES rCSA	1.25 ± 0.29	1.25 ± 0.34	1.32 ± 0.37	1.29 ± 0.34	0.971	0.750
PM FI	0.17 ± 0.21	0.19(0.22)	0.27 ± 0.13	0.25 ± 0.16	0.429	0.719
PM rCSA	0.19 ± 0.07	0.19 ± 0.08	0.19 ± 0.07	0.19 ± 0.07	0.874	0.968
L2						
MF FI	0.38 ± 0.19	0.40 ± 0.19	0.36 ± 0.17	0.38 ± 0.17	0.703	0.682
MF rCSA	0.36 ± 0.13	0.35 (0.15)	0.31 ± 0.09	0.32 ± 0.09	0.332	0.746
ES FI	0.17 (0.22)	0.19 (0.21)	0.14 (0.15)	0.20 ± 0.14	0.349	0.343
ES rCSA	1.26 ± 0.33	1.23 ± 0.33	1.27 ± 0.41	1.23 ± 0.40	0.615	0.704
PM FI	0.16 ± 0.11	0.15 ± 0.10	0.15 ± 0.09	0.15 ± 0.10	0.495	0.932
PM rCSA	0.37 ± 0.14	0.40 ± 0.15	0.37 ± 0.13	0.38 ± 0.13	0.460	0.804
L3						
MF FI	0.40 ± 0.19	0.34 (0.24)	0.37 ± 0.16	0.39 ± 0.17	0.446	0.659
MF rCSA	0.22 (0.17)	0.41 ± 0.10	0.45 ± 0.10	0.48 ± 0.10	**<0.01**	0.239
ES FI	0.22 (0.13)	0.22(0.20)	0.24 ± 0.16	0.24 ± 0.13	0.311	0.894
ES rCSA	0.27 (0.14)	1.09 ± 0.26	1.13 ± 0.29	1.13 ± 0.31	**<0.01**	0.984
PM FI	0.12 (0.14)	0.09 (0.14)	0.12 ± 0.06	0.12 ± 0.08	0.312	0.899
PM rCSA	0.26 (0.25)	0.51 (0.29)	0.60 ± 0.15	0.60 ± 0.15	**<0.01**	0.921
L4						
MF FI	0.37 (0.28)	0.35 (0.19)	0.39 ± 0.19	0.39 ± 0.18	0.405	0.990
MF rCSA	0.28 ± 0.16	0.58 ± 0.16	0.61 ± 0.15	0.63 ± 0.13	**<0.01**	0.749
ES FI	0.34 ± 0.21	0.26 (0.21)	0.28 (0.26)	0.29 (0.22)	0.225	0.386
ES rCSA	0.37 ± 0.20	0.94 (0.28)	0.96 ± 0.24	0.89 (0.21)	**<0.01**	0.396
PM FI	0.10 (0.13)	0.10 (0.12)	0.08 (0.09)	0.10 ± 0.07	0.315	0.317
PM rCSA	0.35 ± 0.19	0.77 ± 0.21	0.80 ± 0.19	0.83 ± 0.19	**<0.01**	0.615
L5						
MF FI	0.46 ± 0.18	0.41 (0.26)	0.45 ± 0.19	0.45 ± 0.18	0.301	0.968
MF rCSA	0.34 ± 0.13	0.64 ± 0.19	0.69 ± 0.16	0.69 ± 0.15	**<0.01**	0.975
ES FI	0.51 ± 0.20	0.47 ± 0.20	0.55 ± 0.20	0.51 ± 0.21	0.319	0.485
ES rCSA	0.48 ± 0.19	0.69 ± 0.28	0.64 ± 0.32	0.57 (0.54)	**<0.01**	0.444
PM FI	0.09 (0.11)	0.09 (0.09)	0.08 (0.07)	0.06 (0.08)	0.395	0.184
PM rCSA	0.46 ± 0.17	0.80 ± 0.27	0.84 ± 0.29	0.84 ± 0.29	**<0.01**	0.989

ALS, amyotrophic lateral sclerosis; LR, lumbosacral radiculopathy; HC, healthy controls; FI, fatty infiltration; rCSA, relative cross-sectional area; MF, multifidus muscle; ES, erector spinae; PM, psoas major; S, symptomatic onset; C, contralateral.

P^1^, *p* value of comparisons between symptomatic onset and contralateral sides in ALS patients.

P^2^, *p* value of comparisons between symptomatic onset and contralateral sides in LR patients.Bold type indicates statistically significant data.

**Figure 4:**
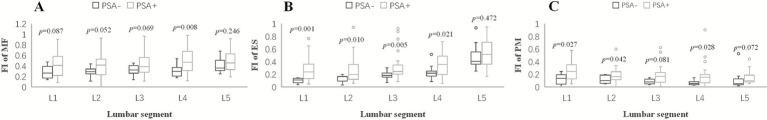
Detailed comparative analysis of the FI of the MF **(A)**, ES **(B)**, and PM **(C)** in ALS patients with pathological spontaneous activity (PSA+) versus those without (PSA-) at the L1–L5 segments.

All 38 ALS patients were divided into two groups based on the presence of pathological spontaneous activity (PSA) in paraspinal muscles. On the symptomatic onset side, the FI of the ES (L1-L4 segments), MF (L4 segment), and PM (L1, L2, L4 segments) was significantly higher in patients with PSA (PSA+) than in those without PSA (PSA-) (Figure 4). Interestingly, no significant differences in the FI of the ES, MF, and PM in LR patients were found between the two groups at the L1-L5 segments (*p* > 0.05).

### Inter-group differences in FI and rCSA

**Figure 5:**
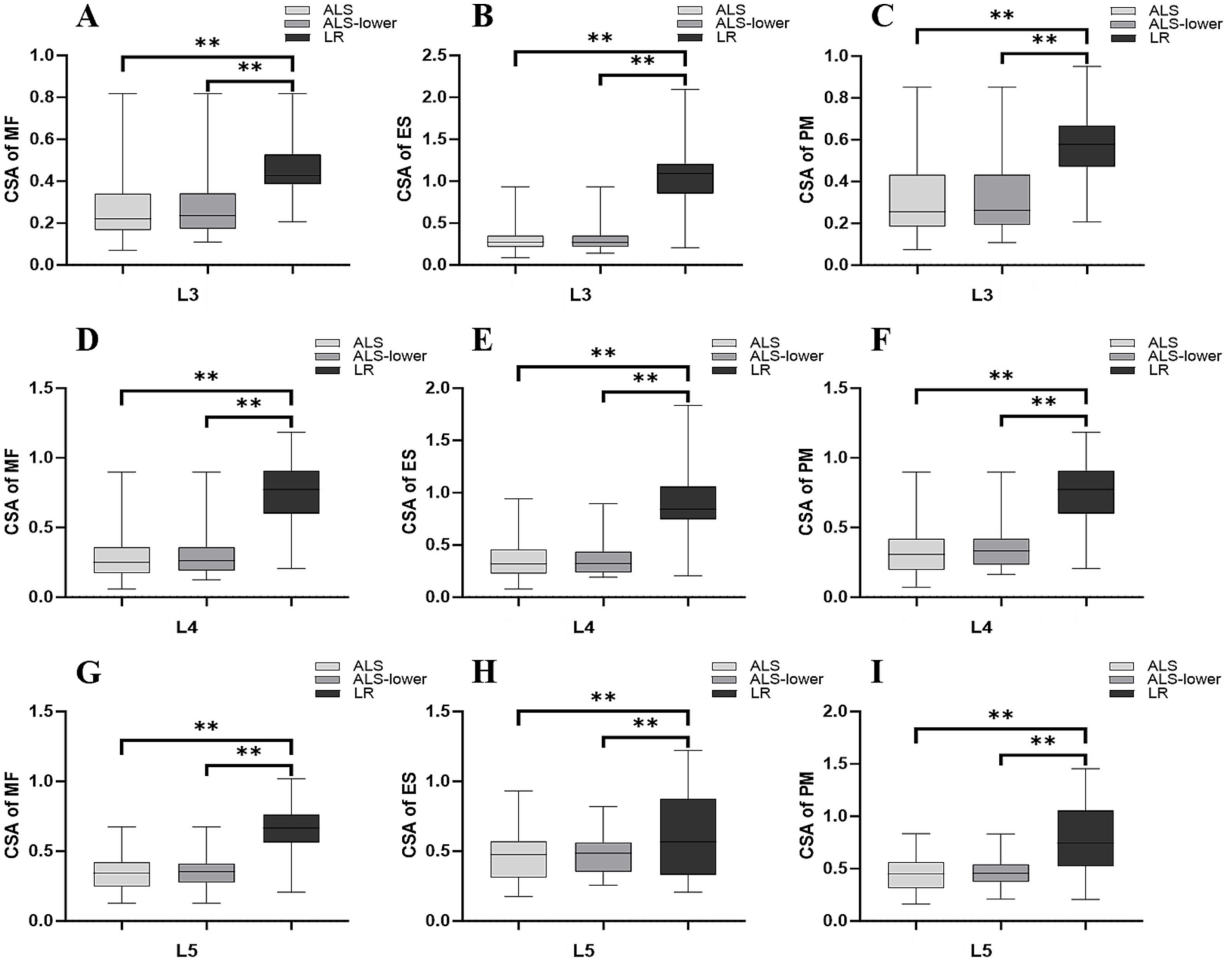
Box-and-whisker plots showing comparative analysis of the rCSA of the MF **(A,D,G)**, ES **(B,E,H)**, and PM **(C,F,I)** on the symptomatic onset side in ALS, lower limb-onset ALS, and LR patients at the L3 **(A–C)**, L4 **(D–F)**, and L5 **(G–I)** segments. **p ≤ 0.01. The horizontal line in the box represents the median. The bottom and top edges of the box represent the 25th and 75th percentiles, respectively. The whiskers (error bars) represent the full range. ALS, amyotrophic lateral sclerosis; FI, fatty infiltration; MF, multifidus muscle; ES, erector spinae; PM, psoas major; FI, fatty infiltration; rCSA, relative cross-sectional area; PSA, pathological spontaneous activity; L, left; R, right.

At the L3-L5 segments, the mean rCSA of the MF, ES, and PM on the symptomatic onset side was significantly higher in LR patients compared to ALS patients (*p* < 0.01). Further comparisons revealed significant differences in the rCSA of the MF, ES, and PM between lower limb-onset ALS patients and LR patients. Overall, significant differences among patients with ALS, lower limb-onset ALS, and LR were observed on the symptomatic onset side at the L3–L5 segments (Table 3; Figure 5). When the discrepancy reached a critical threshold, significant bilateral differences in the rCSA of the MF (L3) and PM (L3–L4) were observed among ALS, lower limb-onset ALS, and LR patients (Table 3). However, no differences were observed in the FI of the MF, ES, and PM between the ALS and LR groups. Interestingly, when these data were normalized by dividing the value of BMI, the symptomatic onset side of ALS patients showed significantly higher FI of the PM compared to LR patients at the L4-L5 segments (L4 segment: 50.00 vs. 44.34, *p* = 0.012; L5 segment: 52.92 vs. 43.13, *p* = 0.042).

**Table 3:** Comparisons of symptomatic onset side and bilateral for MRI data (FI and rCSA) in ALS and LR patients.

Item	ALS (*n* = 38)		ALS-lower (*n* = 26)		LR (*n* = 32)		*P*^1^value		*P*^2^value	
	S	B	S	B	S	B	S	B	S	B
L1										
MF FI	0.37 ± 0.19	0.39 ± 0.18	0.39 ± 0.17	0.42 ± 0.14	0.40 ± 0.18	0.40 ± 0.15	0.49	0.81	0.83	0.60
MF rCSA	0.29 (0.12)	0.59 (0.23)	0.28 (0.12)	0.58 (0.19)	0.29 (0.10)	0.50 (0.25)	0.48	0.08	0.19	0.27
ES FI	0.16 (0.16)	0.17 (0.16)	0.18 (0.15)	0.18 (0.17)	0.17 (0.15)	0.14 (0.13)	0.43	0.44	0.25	0.12
ES rCSA	1.25 ± 0.30	2.50 ± 0.62	1.29(0.43)	2.55 ± 0.55	1.25 ± 0.34	2.61 ± 0.69	0.98	0.51	0.30	0.72
PM FI	0.17 (0.21)	0.18 (0.19)	0.23 ± 0.15	0.23 ± 0.14	0.19 (0.22)	0.26 ± 0.14	0.29	0.12	0.36	0.47
PM rCSA	0.19 ± 0.07	0.38 ± 0.14	0.18 ± 0.06	0.36 ± 0.11	0.19 ± 0.08	0.38 ± 0.13	0.88	0.98	0.60	0.54
L2										
MF FI	0.37 ± 0.19	0.38 ± 0.18	0.37 ± 0.16	0.39 ± 0.14	0.40 ± 0.19	0.37 ± 0.16	0.55	0.72	0.61	0.60
MF rCSA	0.35 ± 0.14	0.72 ± 0.28	0.35 ± 0.15	0.73 ± 0.30	0.35 (0.15)	0.62 ± 0.18	0.27	0.09	0.33	0.10
ES FI	0.17 (0.22)	0.20 (0.18)	0.18 (0.19)	0.20 (0.16)	0.19 (0.21)	0.15 (0.15)	0.27	0.10	0.40	0.06
ES rCSA	1.27 ± 0.34	2.51 ± 0.66	1.32 ± 0.34	2.59 ± 0.66	1.23 ± 0.33	2.51 ± 0.80	0.62	0.99	0.33	0.66
PM FI	0.17 ± 0.11	0.14 (0.14)	0.18 ± 0.12	0.18 ± 0.11	0.15 ± 0.10	0.15 ± 0.09	0.50	0.41	0.21	0.30
PM rCSA	0.37 ± 0.14	0.77 ± 0.28	0.36 ± 0.15	0.75 ± 0.27	0.40 ± 0.15	0.76 ± 0.26	0.46	0.88	0.37	0.90
L3										
MF FI	0.40 ± 0.19	0.40 ± 0.18	0.41 ± 0.14	0.41 ± 0.15	0.34 (0.24)	0.38 ± 0.16	0.47	0.53	0.33	0.40
MF rCSA	0.22 (0.18)	0.81 ± 0.19	0.29 ± 0.15	0.82 ± 0.21	0.41 ± 0.10	0.93 ± 0.19	**<0.01**	**0.02**	**<0.01**	**0.04**
ES FI	0.22 (0.13)	0.22 (0.18)	0.23 (0.13)	0.22 (0.17)	0.22 (0.20)	0.21 (0.20)	0.28	0.30	0.16	0.22
ES rCSA	0.27(0.15)	2.18 ± 0.54	0.27(0.14)	2.18 ± 0.49	1.09 ± 0.26	2.26 ± 0.60	**<0.01**	0.57	**<0.01**	0.59
PM FI	0.12 (0.14)	0.11 (0.13)	0.15 (0.16)	0.13 (0.16)	0.09 (0.14)	0.12 ± 0.07	0.31	0.42	0.11	0.13
PM rCSA	0.26(0.26)	0.99(0.52)	0.26(0.25)	1.04 ± 0.26	0.51(0.29)	1.19 ± 0.28	**<0.01**	**0.03**	**<0.01**	**0.03**
L4										
MF FI	0.37 (0.26)	0.42 ± 0.21	0.45 ± 0.18	0.43 ± 0.17	0.35 (0.19)	0.39 ± 0.18	0.41	0.46	0.20	0.36
MF rCSA	0.28 ± 0.16	1.15 ± 0.31	0.26 (0.19)	1.14 ± 0.33	0.58 ± 0.16	1.34 ± 0.28	**<0.01**	0.20	**<0.01**	0.22
ES FI	0.34 ± 0.21	0.32 ± 0.19	0.34 ± 0.18	0.32 ± 0.17	0.26(0.21)	0.30 ± 0.17	0.23	0.66	0.12	0.64
ES rCSA	0.37 ± 0.20	1.89 ± 0.53	0.37 ± 0.17	1.86 ± 0.44	0.94 ± 0.28	1.89 ± 0.46	**<0.01**	0.96	**<0.01**	0.75
PM FI	0.11 (0.15)	0.10 (0.12)	0.15 (0.15)	0.14 (0.14)	0.10 (0.12)	0.10 ± 0.06	0.20	0.30	0.12	0.10
PM rCSA	0.35 ± 0.19	1.53 ± 0.41	0.36 ± 0.17	1.44 ± 0.33	0.77 ± 0.21	1.63 ± 0.37	**<0.01**	0.27	**<0.01**	**0.04**
L5										
MF FI	0.46 ± 0.18	0.45 ± 0.18	0.46 ± 0.14	0.45 ± 0.15	0.41 (0.26)	0.45 ± 0.18	0.32	0.80	0.20	0.77
MF rCSA	0.34 ± 0.13	1.30 ± 0.37	0.36 ± 0.11	1.24 ± 0.38	0.64 ± 0.19	1.38 ± 0.29	**<0.01**	0.37	**<0.01**	0.12
ES FI	0.51 ± 0.20	0.41 (0.31)	0.52 ± 0.16	0.43 (0.29)	0.47 ± 0.20	0.53 ± 0.19	0.40	0.19	0.25	0.35
ES rCSA	0.47 ± 0.19	1.41 ± 0.56	0.47 ± 0.15	1.36 ± 0.56	0.69 ± 0.28	1.25 ± 0.68	**<0.01**	0.27	**<0.01**	0.51
PM FI	0.09 (0.11)	0.09 (0.10)	0.10 (0.11)	0.10 (0.11)	0.09 (0.09)	0.06 (0.06)	0.40	0.05	0.29	**0.04**
PM rCSA	0.46 ± 0.17	1.59 ± 0.53	0.47 ± 0.14	1.42 ± 0.40	0.80 ± 0.27	1.65 ± 0.59	**<0.01**	0.63	**<0.01**	0.11

ALS, amyotrophic lateral sclerosis; LR, lumbosacral radiculopathy; FI, fatty infiltration; rCSA, relative cross-sectional area; MF, multifidus muscle; ES, erector spinae; PM, psoas major; S, symptomatic onset; B, bilateral.

P^1^, *p* value of comparisons between ALS and LR patients.

P^2^, *p* value of comparisons between ALS with lower limbs onset and LR patients.Bold type indicates statistically significant data.

### Correlations of FI and rCSA with clinical variables

*Partial correlation analysis* was used to assess the correlations between disease severity and paraspinal muscle MRI parameters in ALS patients, controlling for age and BMI. Mild associations were observed between the ALSFRS-lower score and the rCSA of the MF (L1 segment: *r* = 0.415, *p* = 0.015) and PM (L1 segment: *r* = 0.410, *p* = 0.016; L2 segment: *r* = 0.353, *p* = 0.038), as well as the FI of the MF (L1 segment: *r* = −0.393, *p* = 0.021; L2 segment: *r* = −0.366, *p* = 0.031; L3 segment: *r* = −0.346, *p* = 0.041; L4 segment: *r* = −0.380, *p* = 0.024) and ES (L1 segment: r = −0.347, *p* = 0.044; L2 segment: *r* = −0.425, *p* = 0.011) (Table 4).

**Table 4:** Correlations between ALSFRS-r items for lower limbs and MRI data (FI and rCSA).

ALSFRS-lower	FI	rCSA
	*r*, *p*	
L1		
MF	***r* = −0.393, *p* = 0.021**	***r* = 0.415, *p* = 0.015**
ES	***r* = −0.347, *p* = 0.044**	*r* = 0.261, *p* = 0.136
PM	*r* = −0.088, *p* = 0.620	***r* = 0.410, *p* = 0.016**
L2		
MF	***r* = −0.366, *p* = 0.031**	*r* = 0.188, *p* = 0.279
ES	***r* = −0.425, *p* = 0.011**	*r* = 0.110, *p* = 0.528
PM	*r* = −0.082, *p* = 0.640	***r* = 0.353, *p* = 0.038**
L3		
MF	***r* = −0.346, *p* = 0.041**	*r* = 0.243, *p* = 0.160
ES	*r* = −0.260, *p* = 0.132	*r* = 0.257, *p* = 0.136
PM	*r* = −0.106, *p* = 0.544	*r* = 0.247, *p* = 0.153
L4		
MF	***r* = −0.380, *p* = 0.024**	*r* = 0.238, *p* = 0.168
ES	*r* = −0.178, *p* = 0.308	*r* = 0.188, *p* = 0.279
PM	*r* = −0.106, *p* = 0.546	*r* = 0.266, *p* = 0.123
L5		
MF	*r* = −0.294, *p* = 0.086	*r* = 0.337, *p* = 0.147
ES	*r* = −0.291, *p* = 0.090	*r* = 0.257, *p* = 0.136
PM	*r* = −0.101, *p* = 0.562	*r* = 0.389, *p* = 0.201

ALS-FRS-lower, Amyotrophic Lateral Sclerosis Functional Rating Scale revised items for lower limbs; MF, multifidus muscle; ES, erector spinae; PM, psoas major; FI, fatty infiltration; rCSA, relative cross-sectional area.Bold type indicates statistically significant data.

### Consistency and reproducibility of MRI parameter measurements

Intra-observer coefficients of variability for rCSA were <5% for all regions of interest: MF (ICC = 0.986), ES (ICC = 0.968), and PM (ICC = 0.980). Inter-observer reliability was also high for rCSA measurements of the MF (ICC = 0.970), ES (ICC = 0.900), and PM (ICC = 0.905).

## Discussion

In our exploratory study, the rCSA and FI of lumbar paraspinal muscles on T2 MRI were compared between ALS patients and LR patients. We selected lumbar paraspinal muscles because they are responsible for controlling inter-segmental movements and have unique architecture and features (20–22). The MF and ES, two major paraspinal muscle groups, play key roles in lumbar stabilization and mobilization (20, 21, 23). The PM is appropriately positioned to help stabilize the lumbar cylinder, which is critically important for spinal health and contributes to maintaining anterior pelvic tilt balance (24, 25). Importantly, lumbar spine MRI allows for a comprehensive evaluation, including analysis of individual paraspinal muscles at different segments, as well as assessment of deep muscles (7).

Previous studies have investigated changes in the paraspinal muscles of patients with ALS, including fat-fibrotic infiltration, muscle atrophy, alterations in muscle appearance, changes in fiber type composition, and evidence of active or chronic denervation on electrodiagnostic testing (6, 7, 21, 26–30). However, no studies have compared paraspinal muscle changes between lower limb-onset ALS patients and pauci-symptomatic LR patients. In addition, literature on paraspinal muscle MRI in ALS remains scarce (6, 7). Diamanti et al. reported that the median rates of fatty substitution in thoracic paraspinal muscles were not statistically significant on T1-weighted muscle MRI among ALS subgroups and HCs (6). Another MRI study showed a significant difference in the appearance of the PM between ALS patients and LR patients, indicating that T1-weighted MRI of the paraspinal muscles could help distinguish spinal ALS patients from both healthy and pathological controls (7). Recently, Liang et al. (31) evaluated axonal degeneration and muscle changes in the brachial plexus and limb–girdle muscles (LGM) using quantitative MRI in ALS patients with upper extremity onset. Their findings suggested that T1-weighted muscle MRI, while requiring fewer technical resources and capable of providing significant data, is less amenable to routine clinical and EMG assessment (6, 31).

In our study, we demonstrated that the mean rCSA of the MF, ES, and PM was smaller on the symptomatic onset side than on the contralateral side at the L3-L5 segments in ALS patients. However, no differences were observed for the FI of the MF, ES, and PM. Diamanti et al. (6) reported that there was no specific pattern of muscle involvement, and no statistical differences in median FI rates—whether analyzed by region and individually—were found between the left and right sides in ALS patients, rather than between symptomatic and asymptomatic sides. Meanwhile, the same research team reported no significant differences in muscle appearance between the right and left sides in patients with ALS, inflammatory myopathy, and radiculopathy (7). They also found that ALS patients showed less fatty replacement/FI of the PM compared to patients with radiculopathy (7). In contrast to their findings, we found that LR patients had a significantly higher rCSA of the MF, ES, and PM compared to ALS patients at the L3-L5 segments on the symptomatic onset side. However, no differences in the FI of the MF, ES, and PM were observed between the ALS and LR groups. Furthermore, concerning the effect of BMI, imaging data were normalized by dividing the BMI value, and the results showed significantly higher FI of the PM in patients with ALS compared to LR patients at the L4-L5 segments on the symptomatic onset side. This suggests heterogeneity in paraspinal muscle involvement between patients with ALS and those with LR.

In clinical practice, although the diagnosis of ALS may seem straightforward, early differential diagnosis between lower limb-onset ALS patients and pauci-symptomatic LR patients can be challenging. In our cohort, the rCSA of the MF, ES, and PM in lower limb-onset ALS patients was significantly lower compared to LR patients on the symptomatic side at the L3-L5 segments. This suggests that ALS lesions may increase L3-L5 segmental vulnerability compared to other lumbar segments. A possible explanation is that daily repetitive movements of the lumbar spine place greater biomechanical stress on the L3-L5 segments, possibly inducing more chronic impairment of the nerve roots or motor neurons, which is consistent with previous reports (32). On the other hand, ALS patients exhibit lumbar spondylosis more frequently than the general population at comparable ages (33). L3-L5 segmental involvement is consistent with the contiguous distribution along the spinal cord observed in the early stage of the disease, including mechanisms such as ‘prion-like propagation,’ simple diffusion of soluble toxic factors, and cell-to-cell transmission from the onset site (34). Based on these findings, the rCSA emerged as a key indicator for differentiating lower limb-onset ALS patients from LR patients.

Interestingly, differences in the FI of the MF, ES, and PM between the ALS and LR group were not observed in the statistical analysis. The reasons for this are unclear, but it is well documented that variations in muscle fiber type proportions and the lengths of their innervating motor axons may contribute to discrepancies among the involved paraspinal muscles (32). In a previous study, muscle fiber types were analyzed in biopsied specimens using the histochemical method, which showed that Type 2C fibers appear to be more prominent in ALS (35). However, in LR patients, damage to one or more dorsal nerve roots before the fibers converge can cause paresthesia, hypoesthesia, numbness, weakness, and atrophy (36). Muscle atrophy are more susceptible to motor neuron degeneration in ALS than in LR, which could reflect a major contribution of motor neuron degeneration than nerve roots involvement. (II) Muscle volume loss may precede fatty replacement, which can be more sensitively detected by MRI in the lumbar paraspinal muscles. (III) Regarding comparison between the symptomatic and contralateral sides in ALS patients, we found that the symptomatic side had a significantly smaller rCSA of the MF, ES, and PM at the L3-L5 segments, suggesting that muscle atrophy occurs earlier on the symptomatic onset side. Therefore, MRI parameters combined with histomorphometric alterations could be used to investigate pathophysiological mechanisms *in vivo* and to determine whether muscle volume abnormalities are anatomically consistent and sensitive markers in ALS.

Our recent electrophysiological studies demonstrated that PSA (fibrillations and positive sharp waves) in paraspinal muscles correlates with clinical disability in ALS. By stratifying ALS patients according to the presence of PSA in paraspinal muscles, we found that the FI of the ES (L1-L4 segments), MF (L4 segment), and PM (L1, L2, and L4 segments) was significantly higher in patients with PSA than in those without on the symptomatic side, while no differences in the rCSA were observed. In addition, on the symptomatic side, no significant differences in the FI of the ES, MF, and PM were observed between LR patients with PSA and those without at the L1-L5 segments. The divergence in fatty infiltration between ALS patients and LR patients is notable and may be attributable to the distinct disease characteristics and patterns of muscle involvement. Thus, we speculate that higher fatty infiltration in paraspinal muscles with PSA is more specific to ALS. Meanwhile, PSA might be a valuable and sensitive marker for evaluating fatty substitution rather than muscle atrophy in the paraspinal muscles of patients with ALS.

Finally, correlations were observed between disease severity and muscle MRI parameters in ALS patients. Mild associations were found between declines in ALSFRS-lower scores and decreases in the rCSA of the MF and PM. In addition, lower ALSFRS-lower scores were associated with greater FI of the MF and ES. These findings suggest that lumbar spine MRI parameters may serve as a promising tool for the assessment of lower limb involvement in ALS.

### Limitations and future directions

Limitations of this pilot study include the small number of participants and potential influences from premorbid factors such as muscle efficiency. These complexities may not be fully captured by the current MRI assessments. Moreover, the Dixon approach with high-resolution, volumetric acquisitions is needed to investigate more sensitive methodologies for detecting early pathophysiological changes in ALS.

## Conclusion

In summary, muscle atrophy occurs earlier on the symptomatic onset side during disease progression in ALS, and the presence of PSA in paraspinal muscles is a valuable and sensitive marker for evaluating fatty substitution. MRI parameters of paraspinal muscles may be useful for monitoring disease progression in ALS and distinguishing ALS, especially lower limb-onset cases, from pauci-symptomatic LR.

## Data Availability

The raw data supporting the conclusions of this article will be made available by the authors, without undue reservation.

## Glossary

ALS: amyotrophic lateral sclerosis
LR: lumbosacral radiculopathy
HCs: healthy controls
FI: fatty infiltration
rCSA: relative cross-sectional area
MF: multifidus muscle
PM: psoas major
ES: erector spinae
PSA: pathological spontaneous activity
MND: motor neuron disease
ALSFRS-R: ALS functional rating scale-revised
PACS: picture archiving and communication system
IVD: intervertebral disc
AI: artificial intelligence
PIL: Python Imaging Library
ROI: region of interest
ICC: intraclass correlation coefficient
BMI: body mass index
IQR: interquartile range
LGM: limb–girdle muscles
MRI: magnetic resonance imaging
