# Supplementary material for: MRI abnormal patterns of lumbar paraspinal muscles in patients with amyotrophic lateral sclerosis and lumbosacral radiculopathy: a comparative study
Source: Front Neurol. 2026 Mar 17;17:1751139. doi: 10.3389/fneur.2026.1751139 (PMC13064925; doi:10.3389/fneur.2026.1751139)
Supplement: Supplementary file 1 [file Table_1.docx]

**Table 1.** Demographic and clinical features of LR patients

| No. | Age | Gender | Duration(m) | Weakness | Atrophy | PSA | Pandy’s test | MRI |
| --- | --- | --- | --- | --- | --- | --- | --- | --- |
| 1 | 54 | M | 96 | + | + | + | - | - |
| 2 | 59 | F | 15 | + | - | + | - | - |
| 3 | 62 | F | 15 | + | + | + | - | - |
| 4 | 56 | F | 42 | + | - | + | - | + |
| 5 | 39 | M | 6 | - | + | + | NA | + |
| 6 | 23 | M | 1 | + | - | + | - | - |
| 7 | 78 | M | 48 | + | - | - | NA | + |
| 8 | 59 | M | 24 | + | + | - | - | - |
| 9 | 35 | F | 7 | + | + | - | - | - |
| 10 | 69 | M | 22 | - | + | + | - | - |
| 11 | 61 | F | 2 | + | + | - | - | + |
| 12 | 56 | M | 10 | + | + | + | - | - |
| 13 | 61 | F | 2 | + | - | - | - | + |
| 14 | 66 | F | 18 | + | + | + | - | + |
| 15 | 71 | M | 69 | - | + | - | NA | + |
| 16 | 66 | M | 2 | + | + | + | - | - |
| 17 | 63 | M | 14 | + | - | - | - | - |
| 18 | 66 | M | 3 | + | - | - | - | + |
| 19 | 69 | M | 9 | + | + | - | NA | - |
| 20 | 63 | M | 9 | + | - | - | - | - |
| 21 | 35 | F | 7 | + | + | + | - | - |
| 22 | 53 | M | 24 | + | - | - | - | + |
| 23 | 56 | M | 7 | + | - | + | - | - |
| 24 | 54 | F | 3 | + | - | + | - | - |
| 25 | 77 | M | 1 | + | + | + | NA | - |
| 26 | 74 | F | 72 | + | + | + | NA | - |
| 27 | 61 | F | 3 | + | + | + | NA | - |
| 28 | 57 | F | 4 | + | - | + | NA | + |
| 29 | 86 | F | 36 | + | + | + | NA | + |
| 30 | 54 | F | 4 | + | - | + | NA | - |
| 31 | 30 | M | 17 | + | - | - | NA | - |
| 32 | 63 | M | 5 | + | - | + | - | + |

NA: Not applicable
